# Ophthalmological Findings in Mucopolysaccharidoses

**DOI:** 10.3390/jcm8091467

**Published:** 2019-09-14

**Authors:** Shizuka Tomatsu, Susanne Pitz, Ulrike Hampel

**Affiliations:** 1 Department of Ophthalmology and Visual Sciences, University of Illinois at Chicago, Chicago, IL 60607, USA; 2 Ophthalmic Clinic, Bürgerhospital, 60318 Frankfurt, Germany; s.pitz@buergerhospital-ffm.de; 3 Department of Ophthalmology, University Medical Center, Johannes Gutenberg University, 55131 Mainz, Germany; uli.hampel@gmail.com

**Keywords:** mucopolysaccharidosis, lysosomal storage disorder, ophthalmology, ocular manifestations, corneal clouding

## Abstract

The mucopolysaccharidoses (MPS) are a heterogenous group of lysosomal storage disorders caused by the accumulation of glycosaminoglycans (GAGs). The accrual of these compounds results in phenotypically varied syndromes that produce multi-organ impairment with widespread systemic effects. The low incidence of MPS (approximately 1/25,000 live births) in conjunction with the high childhood mortality rate had limited the availability of research into certain clinical features, especially ocular manifestations. As the recent successes of hematopoietic stem cell transplantation (HSCT) and enzyme replacement therapy (ERT) have greatly increased life expectancy in these patients, they have served as a focal point for the transition of research towards improvement of quality of life. Ophthalmological findings in MPS include corneal clouding, glaucoma, optic neuropathies, and retinopathies. While corneal clouding is the most common ocular feature of MPS (especially type I, IVA, and VI), its response to HSCT and ERT is minimal. This review discusses known eye issues in the MPS subtypes, diagnosis of these ocular diseases, current clinical and surgical management, noteworthy research progress, and ultimately presents a direction for future studies.

## 1. Introduction

The mucopolysaccharidoses (MPS) are a heterogenous group of lysosomal storage disorders caused by the intra and extracellular accumulation of glycosaminoglycans (GAGs). A deficiency of various enzymes along these polysaccharide breakdown pathways results in the systemic manifestations of this disease (Table 1) [1,2,3]. While the majority of subtypes are inherited in an autosomal recessive manner, type II is X-linked [1]. Observed symptoms include growth restriction, cognitive impairment, skeletal abnormalities, structural cardiac valvular dysfunction, visual impairment, hearing loss, and upper airway obstruction [1].

**Figure 1:**
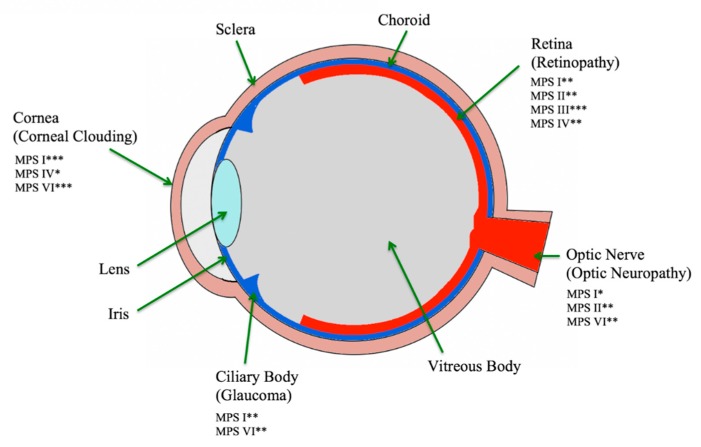
Sagittal view of eye listing area of effect in MPS (mucopolysaccharidosis) subtypes. Modified from [2,3]. * mild, ** moderate, *** severe.

Low incidence and a high childhood mortality rate have led to limited research into the non-fatal manifestations. In recent years, the introduction of hematopoietic stem cell transplantation (HSCT) and enzyme replacement therapy (ERT) has greatly increased the lifespans of these patients, but the greatest effect is achieved when treatment is initiated within the first 16 months of life [1,2,4,5]. The clinical manifestations involving multiple organ systems can present as late as three years of age, not uncommonly delaying diagnosis and reducing the effectivity of therapies [6]. Ophthalmologists play a vital role in diagnosis and management through the early detection of ocular symptoms (Figure 1) [1,2,4,5]. This review aims to illustrate the ophthalmological manifestations among the MPS subtypes, provide diagnostic pearls, current management, and the state of active research into this topic.

## 2. Methods

Our literature review enrolled English language PubMed-listed publications and reports of cases diagnosed with MPS from 1981 to 2018. Search terms included: ‘Mucopolysaccharidosis’, and ‘ocular’, ‘ophthalmological’, ‘cornea’, ‘retina’, or ‘eye.’ We identified 61 articles/reports containing information about 2119 ocular cases.

## 3. MPS I

### 3.1. Natural History of MPS I

Mucopolysaccharidosis I is an autosomal recessive disease caused by α-l-iduronidase deficiency (Table 1). This enzyme is required for the hydrolysis of non-reducing terminal α-l-iduronide glycosidic bonds in two GAGs—dermatan sulfate (DS) and heparan sulfate (HS). The enzyme deficiency leads to the accumulation of DS and HS. There are three phenotypic heterogenetic subtypes—Hurler, Hurler–Scheie, and Scheie, with Hurler being the severe form and Scheie being the mildest [7,8]. The incidence of the attenuated form is 1 out of 115,000 to 500,000 live births, and the severe form 1 out of 100,000 [2,8]. The first symptoms in Hurler syndrome occur within the first two years of life whereas in Hurler–Scheie and Scheie syndromes, onset occur between the ages 3 and 7 years, and 5 and 13 years, respectively [8]. Without systemic treatment, most patients with Hurler die within the first decade of life, while those with Hurler–Scheie and Scheie have longer lifespans due to their attenuated symptoms [8]. Lifespan is vastly dependent on the age of symptom onset and the rate of disease progression [8]. Despite these differences in disease severity, ocular manifestations are seen in all three phenotypes [1,8].

### 3.2. Ocular Issues in MPS I

**Figure 2:**
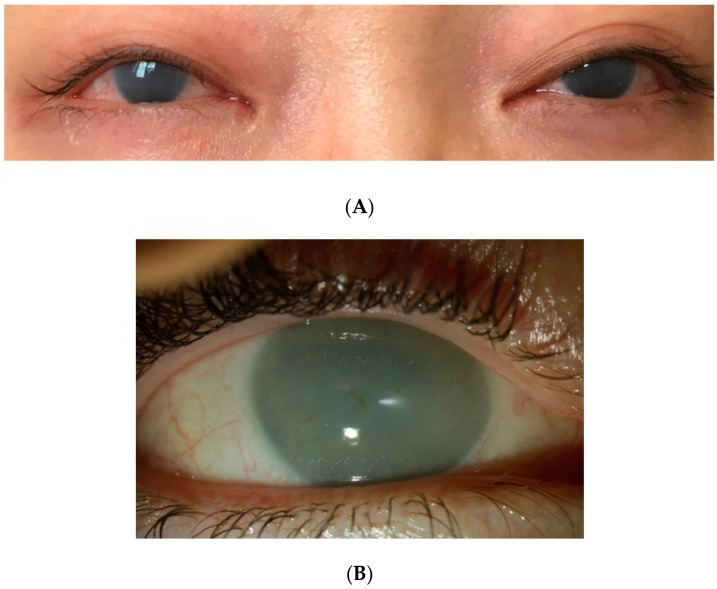
Corneal clouding in MPS I. The top picture (**A**) shows a 19-year-old patient. The bottom picture (**B**) shows a 38-year-old patient. No systemic treatment for both patients.

Corneal clouding, which can develop within the first year of life, is observed in the majority of MPS I patients, regardless of the syndrome [7,8]. Light is refracted in a healthy transparent cornea to project an image onto the retina. When the cornea is translucent or opaque, as in most MPS I cases, light scatters, resulting in blurry or even no images arriving at the retina [5]. Although GAGs deposit in all layers of the cornea, the opacity, often described as a diffuse ground glass appearance, is caused by the disruption of normal collagen alignment in the corneal stroma (Figure 2) [4,9,10]. In addition to the visual obstruction caused by the corneal opacity, GAG deposits increase the corneal thickness, which leads to difficulty in diagnosing and managing glaucoma [1,5].

A tool to diagnose and monitor glaucoma includes measuring the intraocular pressure (IOP). Changes in corneal thickness can influence IOP measurements, and corneal clouding can prevent an accurate visualization of the optic nerve and the corneal-scleral angles, all necessary in glaucoma diagnosis [1,2,4,5]. A cross-sectional study of MPS I children showed that IOP measurements need to be corrected to avoid unnecessary medical or surgical glaucoma treatments [11]. This study showed that both IOP and corneal hysteresis were falsely elevated in MPS I, due to the high corneal resistance [11]. When accounting for corneal thickening, all eyes were within normal limits of IOP, and no evidence of glaucomatous cupping was detected on imaging [11]. Both open and closed angle glaucoma can still occur due to the GAG accumulation in the trabecular meshwork [1,11]. Optic atrophy and swelling may occur in Hurler and Hurler–Scheie syndromes. In the Scheie phenotype, disk swelling, and macula edema-like changes have been reported, but optic atrophy and swelling are less common [1,12]. Refractive errors, especially hypermetropia, are also common in MPS I due to the inflexible corneal curvature and the inelastic shortened axial length of the sclera, all relating to GAG storage [13,14,15]. Ocular motility abnormalities are common, especially exotropia, secondary to decreased corneal opacity and GAG deposition in the extra-ocular muscles [1,9,10]. Other case reports and studies note retinopathies in all three MPS I syndromes [1,10,16,17]. Visual changes from optic neuropathies and retinopathies may go unnoticed due to the overlying corneal clouding.

## 4. MPS II

### 4.1. Natural History of MPS II

Mucopolysaccharidosis II (Hunter syndrome) is the only X-linked disease among the MPS family (Table 1). The deficiency of iduronate-2-sulfatase, an enzyme that catalyzes sulfate group removal, results in the buildup of DS and HS [18]. The prevalence of Hunter syndrome is about 1 in 100,000 to 170,000 live male births, with the highest prevalence seen in Ashkenazi and Oriental or Sephardic Jews living in Israel [2,18]. Although rare, MPS II females are typically afflicted with the reduction of iduronate-2-sulfatase as opposed to the complete lack of the enzyme in males. Occasionally, a heterozygous female will be fully afflicted due to skewed X-chromosomal inactivation, rendering a non-functional normal allele [19]. The female clinical symptoms are attenuated in comparison to males [18,19]. Phenotypic features are often unnoticed at birth but become more obvious by the age of 2 to 4 years old [18]. Most patients die in their second decade of life if untreated, and only the mild cases are seen to live into their fifth or sixth decade [19]. Although ocular manifestations are not the most prominent features of MPS II, there is a higher incidence of the posterior chamber than anterior chamber conditions (Figure 1) [5,18,19].

### 4.2. Ocular Issues in MPS II

**Figure 3:**
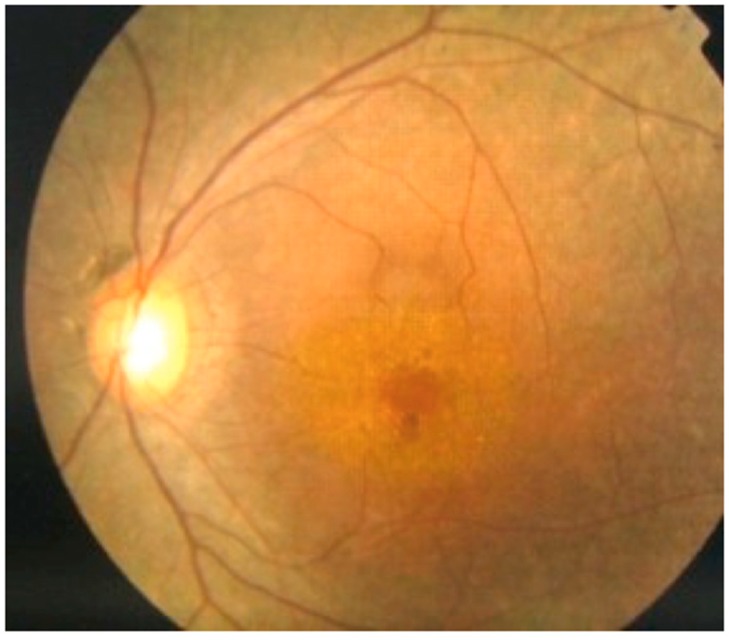
Retinitis pigmentosa in MPS II. Fundoscopy showing retinitis pigmentosa in a 39-year-old MPS II patient.

Corneal clouding, aside from the rare cases, is not common in MPS II [1,18,20]. The most common ocular findings are hypertelorism and exophthalmos, which can lead to chronic conditions related to corneal overexposure (i.e., corneal abrasions, keratoconjunctivitis, etc.) [1]. Hunter patients also have a moderate incidence of optic nerve abnormalities and retinopathy [4,12]. Although glaucoma is rarely seen, increased pressure at the optic nerve, due to the scleral GAG deposition and the subsequent thickening, leads to chronic disc elevation without increased intracranial pressure [19]. Retinopathy is variable, depending on the severity of the disease, but electroretinograms have supported many MPS II cases with nyctalopia (Figure 3) [4,18,19,21].

## 5. MPS III

### 5.1. Natural History of MPS III

Mucopolysaccharidosis III (Sanfilippo syndrome) is an autosomal recessive disease with four subtypes, A, B, C, and D, with deficiencies in heparan-*N*-sulfatase, α-*N*-acetylglucosaminidase, α-glucosaminide acetyltransferase, and *N*-acetylglucosamine-6-sulfatase, respectively, all leading to the accumulation of HS (Table 1). The four subtypes are phenotypically difficult to distinguish [1,2]. Unlike the other MPS, Sanfilippo primary affects the central nervous system with mild somatic effects—classic findings include hyperactivity and neurocognitive-behavioral issues [22]. While MPS IIIA and MPS IIIB remain the most common subtypes, the combined incidence for all subtypes is about 1 in 70,000 newborns [2,22]. Patients are often diagnosed when language delay, behavioral issues, and hyperactivity are noticed between the ages of 2 and 6 years old [22]. By age 10, up to 60% of MPS III patients experience seizures [1]. Insidious progressive gait disorders and pyramidal signs after the first decade of life often lead to a vegetative state and death by their early 30s [22].

### 5.2. Ocular Issues in MPS III

The most prominent ocular condition seen in Sanfilippo syndrome is retinopathy [2,4,5,23]. Heparan sulfate aggregations within the retinal pigment epithelial cells and in the photoreceptor matrix lead to progressive photoreceptor loss, retinal dysfunction, and retinal degeneration [4,5]. Many MPS III patients demonstrate moderately to severely affected electroretinograms, showing reduction in b-waves on dark adaptation due to the decreased rod-mediated responses [4]. Pigmentary changes in the retina are often recognized in these patients [1,2]. Clinically, patients often notice nyctalopia and decreased vision [5]. Corneal opacification is not commonly associated with MPS III [1,2,4,5,24]. Other ocular issues include glaucoma, optic atrophy, and swelling of the optic disk although these are not seen as often as retinal pathologies [1,2,4,5,12].

## 6. MPS IV

### 6.1. Natural History of MPS IV

Mucopolysaccharidosis IV (Morquio syndrome) is an autosomal recessive disease that is split into two different subtypes, MPS IVA and MPS IVB, with deficiency of *N*-acetylgalactosamine-6-sulfatase (GALNS) and β-galactosidase, respectively (Table 1) [1,2]. Both Morquio syndromes result in the accumulation of keratan sulfate (KS), in addition to chondroitin-6-sulfate (C6S) for type A, especially in bone, cartilage, heart valves, and cornea [2,25]. Incidence varies depending on the ethnic population and country but can be as common as 1 in 76,000 births in Northern Ireland to 1 in 640,000 births in Western Australia [25]. The accumulation of KS and C6S, predominantly affecting the skeletal system and chondrocytes, begins prenatally, with patients showing symptoms after their first year of life [2,25]. Depending on the severity of the disease, patients vary in skeletal abnormalities (prominent forehead, short neck, instability of the neck, prominent chest, hypermobile joints, kyphoscoliosis, waddling gait, knock-knee) and consequent mobility deficits. By their teenage years, many are wheelchair bound [25]. Abnormal chondrogenesis is one of the primary issues; patients often die from respiratory obstruction and cervical spinal cord complications between their second to third decade of life, especially if untreated [25].

### 6.2. Ocular Issues in MPS IV

Common ophthalmological manifestations are diffuse corneal clouding and refractive errors [26,27]. Although the corneal opacification is not as severe as in MPS I and MPS VI, patients can still experience photosensitivity and may notice worsening corneal haze with age [16,26]. Refractive lenses are often used for astigmatism, myopia, and hyperopia [26]. Due to their shallow orbits from abnormal bone formation, corneal exposure keratopathy from pseudoexophthalmos can occur. Scattered cases of cataracts, glaucoma, retinopathy, optic disc atrophy, and optic disc swelling exist [12,26,27].

## 7. MPS VI

### 7.1. Natural History of MPS VI

Mucopolysaccharidosis VI (Maroteaux–Lamy syndrome) is an autosomal recessive disease caused by a deficiency in *N*-acetylgalactosamine-4-sulfatase, leading to the accumulation of DS and chondroitin-4-sulfate (C4S) (Table 1). The incidence is about 1 case per 250,000 to 600,000 live births, depending on the ethnic population [2]. Patients show signs of significant growth retardation by 24 months of age, and depending on the severity of the disease, mortality ranges from late teens to the fifth decade of life. Cardiopulmonary complications are the primary cause of death [28].

### 7.2. Ocular Issues in MPS VI

**Figure 4:**
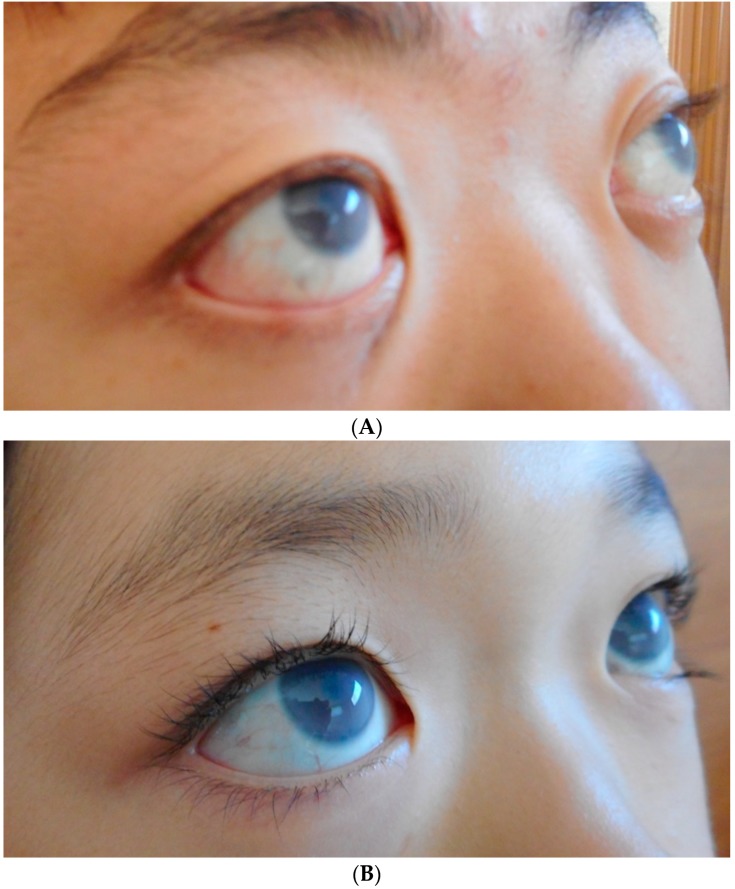

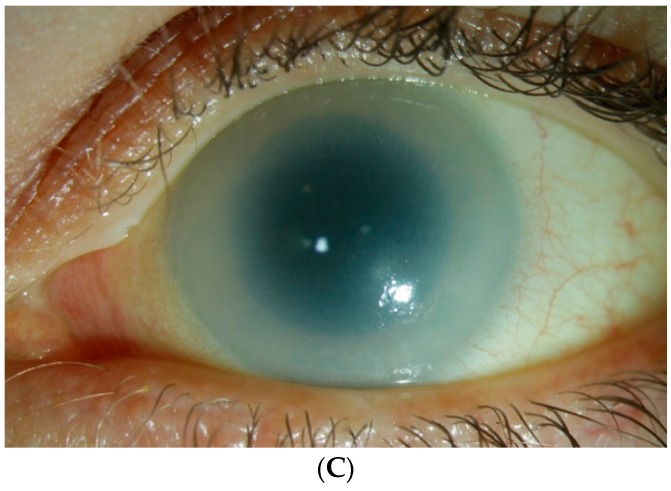
(**A**) Corneal clouding in MPS VI. (**A**) shows a 17-year-old male with enzyme replacement therapy (ERT). (**B**) shows an 11-year-old female with ERT. (**C**) shows a 38-year-old female with no systemic treatment.

Corneal opacity with corneal thickening is common in MPS VI (Figure 4) [11,16]. Much like MPS I, IOP measurements may be inaccurate due to the increased corneal hysteresis, which leads to difficult detection and management of glaucoma [5,11]. Optic nerve abnormalities are also common [12,29]. GAGs infiltrate within the optic nerve and cause nerve swelling. Patients who have GAG-induced obstructive hydrocephalus can also acquire papilledema [5]. Ocular motility is another common issue, which can lead to strabismus and amblyopia [4,5]. MPS VI is not associated with retinopathy [1,2,28].

## 8. MPS VII

### 8.1. Natural History of MPS VII

Mucopolysaccharidosis VII (Sly syndrome) is an autosomal recessive disease caused by β-galactosidase deficiency (Table 1). This results in the partial degradation and fragment accumulation of C4S, C6S, DS, and HS. The incidence is about 1 in every 300,000 to 2,000,000 live births [30]. Variation in disease severity remains one of the primary predictors of mortality, ranging from prenatal death (severe cases) to survival into the fifth decade of life (mild cases with residual enzymatic activity) [31]. Rarer forms of MPS VII result in stillbirth or hydrops fetalis [2,31]. Due to the rarity of this disease, MPS VII is not as well understood as the other MPS types [30].

### 8.2. Ocular Issues in MPS VII

The most common eye issue is corneal opacification, though typically not as severe as MPS I or VI [30,32]. Reports of optic nerve abnormalities exist; however, the incidence of glaucoma and retinopathy remain unknown thus far [12,31].

## 9. MPS IX

### 9.1. Natural History of MPS IX

Mucopolysaccharidosis IX (Natowicz syndrome) is the rarest form of the MPS family with very little data available [2]. The syndrome is caused by hyaluronidase deficiency leading to hyaluronan accumulation. The first case report was in 1996 of a 14-year old girl with a history of mild craniofacial abnormalities, recurrent otitis media, soft tissue masses, and self-limiting painful periarticular masses occurring after febrile illness or physical exertion [33]. Other cases include three siblings with isolated joint pathology, cleft palate, knee or hip pain and swelling, and recurrent otitis media [34]. Upon further investigation, all of these cases had hyaluronidase deficiency, leading to the accumulation of hyaluronan [33,34].

### 9.2. Ocular Issues in MPS IX

No cases to date have reported ocular manifestations in MPS IX. This may also be due to the lack of data availability, as well as the low prevalence of the disease [2,5].

## 10. Ophthalmological Management and Intervention

The introduction of systemic therapies has prolonged lifespans for patients with MPS, allowing for a renewed focus into quality of life as it pertains to management. Clinical guidelines were recently created to address the early diagnosis and intervention of ocular issues [10].

### 10.1. Refractive Errors

Refractive errors, especially hypermetropia and astigmatism, are common, but traditional spectacles do not benefit all patients due to comorbid ocular disorders [9,35]. Visual acuity should be assessed at both distance and near vision, using different techniques according to each age group [9,10]. For preschool ages—Teller acuity cards and Kays pictures; for school-aged children—E test and ETDRS acuity test. Refraction with an autorefractor or a retinoscope is highly recommended after cycloplegia with cyclopentolate and phenylephrine. In cases of strabismus, stereopsis assessment and an accommodative fixation target should be utilized [10]. Photochromatic lens can benefit symptoms of photophobia [9,35].

### 10.2. Corneal Clouding

Corneal clouding is one of the major issues in MPS I and VI, and to some extent for MPS IVA and VII [9,11,30,36]. The buildup of GAGs in the cornea not only obstructs the vision for these patients but also interferes with accurate ophthalmological exams for conditions, such as glaucoma, optic neuropathies, and retinopathies [1,2,4,5]. A slit lamp exam with photographs is useful in assessing the degree of the corneal opacification over time, and to examine any surface changes or vascularization [10]. Couprie et al. suggests a semi-objective grading method using the visibility of the iris and anterior chamber details (ranging from mild to severe) [37]. More precise and objective methods using the iris camera and Pentacam may be difficult to apply in all clinical settings [38,39]. Although not routinely used, in vivo confocal microscopy analyzes microscopic corneal transformations, and can aid a better understanding of a patient’s corneal health [10]. Currently, the only treatment for corneal opacification is a corneal transplant [2,5]. Both penetrating keratoplasty (PK) and deep anterior lamellar keratoplasty (DALK) have been conducted in MPS patients [5,40]. A recent retrospective study with MPS I, IV, and VI patients who underwent either PK or DALK showed that 94% of the patients had clear grafts at their last follow-up visits [40]. PK is a full thickness corneal transplant, which includes the removal of the corneal epithelium, stroma, and endothelium, whereas DALK only replaces the epithelium and stroma. In MPS cornea, the majority of GAG deposition appears in the epithelium and the stroma, with the endothelium unaffected until later stages of the disease. A full thickness corneal transplant is unnecessary for many MPS patients whose corneal endothelium is still healthy. The intact Descemet’s membrane (posterior to the stroma and anterior to the endothelium) may act as a protectant for the involvement of the endothelial layer, as well as prevention of GAG re-deposition in the stroma post-transplant. Preserving the patient’s endothelial layer also negates the risk of endothelial graft rejection. Therefore, DALK is often recommended over PK [5,41]. Another suggestion includes combining DALK with limbal stem cell transplantation. By introducing healthy limbal epithelial cells to the host, this may delay or even prevent corneal clouding after keratoplasty [5,42]. Attention must be taken to weigh the benefits for a patient with corneal clouding and concomitant retinopathy or glaucoma before deciding on a surgical intervention [5,10].

### 10.3. Keratoconjunctivitis Sicca

Dry eye syndrome is prevalent for patients who have undergone HSCT [43]. In these cases of keratoconjunctivitis sicca, in addition to decreasing corneal exposure, topical lubricants are recommended, with topical steroid and/or topical cyclosporine for severe cases. Topical lubricants are also recommended in pseudoexophthalmos [9].

### 10.4. Glaucoma

Glaucoma can occur in all MPS types in varying frequency secondary to the deposition of GAGs in the anterior chamber structures. Similar to other open-angle glaucoma pathophysiology, accumulation of the GAGs in the trabecular meshwork and aqueous outflow pathways can lead to glaucomatous changes in the optic nerve. Closed-angle glaucoma may be induced by GAG depositions in the peripheral cornea, other anterior chamber structures, and cystic changes in the ciliary body. Visual field exams via finger counting (although not the most accurate method), Humphrey field analyzer, and Goldmann visual field perimetry are utilized, depending on the patient’s age and intellectual ability [10]. To conduct a thorough glaucoma evaluation, techniques outside of the normal gonioscopy and slit lamp examinations are used to better visualize the anterior chamber and the optic nerve [4,5,11]. Ultrasound biomicroscopy and anterior segment optical coherence tomography (OCT) provide detailed views of the anatomy behind the potentially hazed cornea, aiding in the diagnosis and treatment of glaucoma [5]. As discussed earlier, MPS patients may have falsely elevated IOP due to the increased rigidity of their cornea. OCT assists in visualization of the corneal layers, thickness of the retina, photoreceptor layers, retinal nerve fiber layers (RNFL), and the optic nerve. Goldmann applanation tonometry and the ocular response analyzer have less dependence on the cornea properties and may be more reliable when measuring the IOP [44,45,46]. Another tool that helps bypass the issue of the opaque cornea during a trabecular surgery is the microendoscope [47]. For MPS patients, it is crucial for ophthalmologists to discern the diagnosis of glaucoma through extra caution and noting that the measured IOP reading may be inaccurate. 

### 10.5. Retinopathy

Posterior chamber diseases require a complete fundus exam to look for the optic nerve and retinal pathologies. In cases where this is limited by a clouded cornea and photophobia, fundus photography and echography may be the best solutions [10,44]. Fundus photography can produce a better image than expected, even if the manual visualization of the retina is obstructed by the corneal opacity. Echography aids in examining the vitreous and the retina, and an A-scan ultrasound is useful for axial length measurement. Electroretinography is useful in detecting rod-cone degeneration [5,10]. Although MPS III patients have the highest incidence of retinopathies, the severity of their neurocognitive and behavioral issues warrants an individual-based clinical decision. A full retinal workup may not change the overall management [4,22].

### 10.6. Anesthesia Issues

Examinations performed under anesthesia may be required depending on the degree of mental disability and cooperation from the patient. However, MPS patients often have cardiopulmonary comorbidities with craniofacial structural abnormalities, enlarged adenoids, tonsils, tongue, and laryngopharynx—all of which complicate airway management. Aside from MPS III, emergency tracheostomy due to failed tracheal intubations is possible in all MPS types, leading to a high-risk surgery [48]. Although interventions for the eye conditions are available, examination and treatment are dependent on the individual’s ability to remain still and/or the individual’s tolerance to anesthesia, requiring a multi-disciplinary team.

## 11. Hematopoietic Stem Cell Transplant and Enzyme Replacement Therapy

Hematopoietic stem cell transplant (HSCT) was first performed on a 9-month-old with Hurler syndrome in 1980 as a method to decelerate disease progression [49]. In HSCT, matched donor bone marrow or umbilical cord stem cells with normal enzymes are given to the recipient [2,50]. These normal cells travel throughout the recipient’s body, in the hope that the enzymes will penetrate the recipient tissues [2].

A retrospective international multi-center study from 2015 measured both primary endpoints and secondary endpoints of pre- and post-HSCT from 217 MPS I Hurler patients [50]. One of the major primary endpoints included developmental outcomes after the treatment. The study showed that receiving HSCT in childhood led to better cognitive and developmental outcomes, especially among those who received it before 16 months of age. Ocular issues were also measured as a secondary endpoint in this study. Data showed that 97.6% of the patients had corneal clouding before HSCT. After HSCT, the majority (73.8%) were either stabilized or showed improvement in their cornea. The progression of corneal clouding was measured by the occurrence of corneal transplantation. Keratoplasty only happened in 9.8% of the total patients at a median follow-up of 11.1 years post-HSCT. Glaucoma drops were necessary for 11 patients and cataracts were only observed in patients who received total body irradiation before their HSCT, a practice which was discontinued in 2002 due to adverse effects (i.e., hypothyroidism, cataract, neurodevelopment and growth issues) [50]. 

Other studies have also compared the ocular presentation of MPS patients at follow-up and found that HSCT may stabilize or improve corneal opacification [49,50,51]. In a long-term follow-up study after 5 to 6 years post-HSCT, the majority of patients had stabilized or showed improved corneal clouding, but a significant number of patients had worsening retinal dysfunction [52]. Many of the MPS patients in these studies were MPS I Hurler, with sparse numbers of MPS III and VI patients [52,53]. Overall, the effects of the HSCT are most significant if given to the patient before the age of two, which preserves cognitive development and stabilizes or improves corneal clouding, especially in MPS I patients [49,50]. However, stem cell transplant is not without risks, as a European retrospective analysis reported the mortality rate as 15% and survival rate with engraftment as 56% [54]. A safer approach, such as enzyme replacement therapy (ERT), has been available to MPS patients since 1999 [2,3,50].

Currently, there are four available ERTs—Aldurazyme^®^ (BioMarin Pharmaceutical Inc., Novato, CA, USA) for MPS I, Elaprase^®^ (Shire Human Genetic Therapies Inc., Cambridge, CA, USA) for MPS II, Vimizim^®^ (BioMarin Pharmaceutical Inc., Novato, CA, USA ) for MPS IVA, and Naglazyme^®^ (BioMarin Pharmaceutical Inc., Novato, CA, USA) for MPS VI [17].

This low-risk and well-tolerated treatment provides a tremendously positive effect on hepatomegaly and joint range of motion [55,56]. Unfortunately, the effect of ERT on ocular manifestations are limited and variable [53]. When ocular outcomes of post-treatment are mentioned, corneal opacity is often the only measure discussed for MPS I and VI. One canine study compared corneal GAG accumulation in low-dose ERT, high-dose ERT, and no treatment groups. Only the high-dose ERT canine group showed significantly less GAG accumulation in their cornea [57]. Experience with human patients has revealed that ERT has little to no effect on the ocular manifestations of MPS. This is thought to be in part due to the retina–brain barrier and the avascular nature of the cornea, resulting in the difficulty of ERT to affect eye pathology effectively. In some MPS I patients, combining HSCT and ERT is an appropriate and safe method of treatment. The decision is dependent upon the neurocognitive developmental stage and the age of the patient being treated [58,59].

One study attempted a direct approach by intrastromally transplanting umbilical mesenchymal stem cell (UMSC) isolated from neonatal umbilical cords into MPS VII murine corneas at 1, 2, and 3 months of age [31]. In all three age groups, the UMSC-treated mice showed a significant decrease in corneal haze, corneal thickness, and the overall GAG accumulation in the cornea when compared to the non-treated eyes. Of note, the post-UMSC treatment mice in the 1- and 2-month groups both showed synonymous levels of GAG in the cornea compared to the control non-MPS littermates. Accumulated GAGs within the corneal lysosomes were metabolized in the murine cornea via exosomes from the UMSC, which induced a cell–cell trafficking of the deficient enzyme to the recipient corneal cells. Lysosomes were decreased both in number and size in the post-UMSC-treated corneas, again strengthening the hypothesis that the defective enzyme in MPS VII, β-galactosidase, was delivered from the UMSC to the diseased cornea. These studies have shown that a more direct approach is necessary to treat ocular pathologies in MPS rather than systemic treatments, such as HSCT and ERT. Future studies may benefit more by focusing on eye drops and direct stem cell transplants into the eye.

## 12. Corneal Clouding Theories

The cornea is comprised of the epithelium and endothelium, with a thicker layer of stroma in between. Thickest at the central cornea, the stromal layer is organized predominantly by type I collagens and proteoglycans, a macromolecule made up of a protein core, with GAG side chains composed of either keratin (KS) or dermatan sulfate (DS). Keratin proteoglycans regulate the diameter of collagen fibrils while dermatan proteoglycans control interfibrillar spacing and collage adhesion. Heparan sulfate (HS) is synthesized by corneal epithelial cells but plays a minor role in the cornea [60]. 

While the exact mechanism underlying corneal clouding remains elusive, one prominent theory involves defects in the organization of type 1 collagens. Decorin is a dermatan proteoglycan that regulates collagen fibrogenesis, and defects in the *decorin* gene result in diffuse corneal clouding with decreased corneal transparency [61].

Differences in corneal collagen expression were evaluated in patients with MPS I in comparison to age-matched controls from healthy corneas. The mucopolysaccharide deposition in MPS corneas led to thicker stromal layers, with up to 30-fold increases in type I collagens and notable increases in other collagen types. This increased collagen was noted to be significantly disorganized throughout the cornea. Additionally, α-smooth muscle actin expression, an indicator of stromal cell conversion into myofibroblasts, was increased in MPS I corneas. This conversion is associated with an increase in corneal collagen synthesis, seen in corneal injuries and opacity. It remains unclear if the myofibroblast conversion directly affects GAG deposition or if the collagen organization is affected [62].

Normal age-related corneal changes have also assisted in the understanding of how GAGs affect corneal transparency. When comparing non-diseased young human cornea with aging human cornea, there was a significant age-related GAG change in the two age groups. There was a 30% increase of total GAGs in the older subjects than in the younger subjects. Specifically, there was a higher ratio of heavily sulfated GAGs, such as DS, HS, and KS, in the older subjects. This age-related change in GAGs is hypothesized to be due to decreased antioxidant enzymes in older eyes, increasing the risk of oxidative stress and reducing the healing of corneal damage [63].

## 13. Lack of Corneal Clouding in MPS II

As mentioned above, DS is believed to play a significant role in the pathophysiology of corneal clouding. This GAG accumulates in MPS I, II, VI, and VII, with each of those exhibiting corneal clouding except type II [5,11,30,36]. 

**Figure 5:**
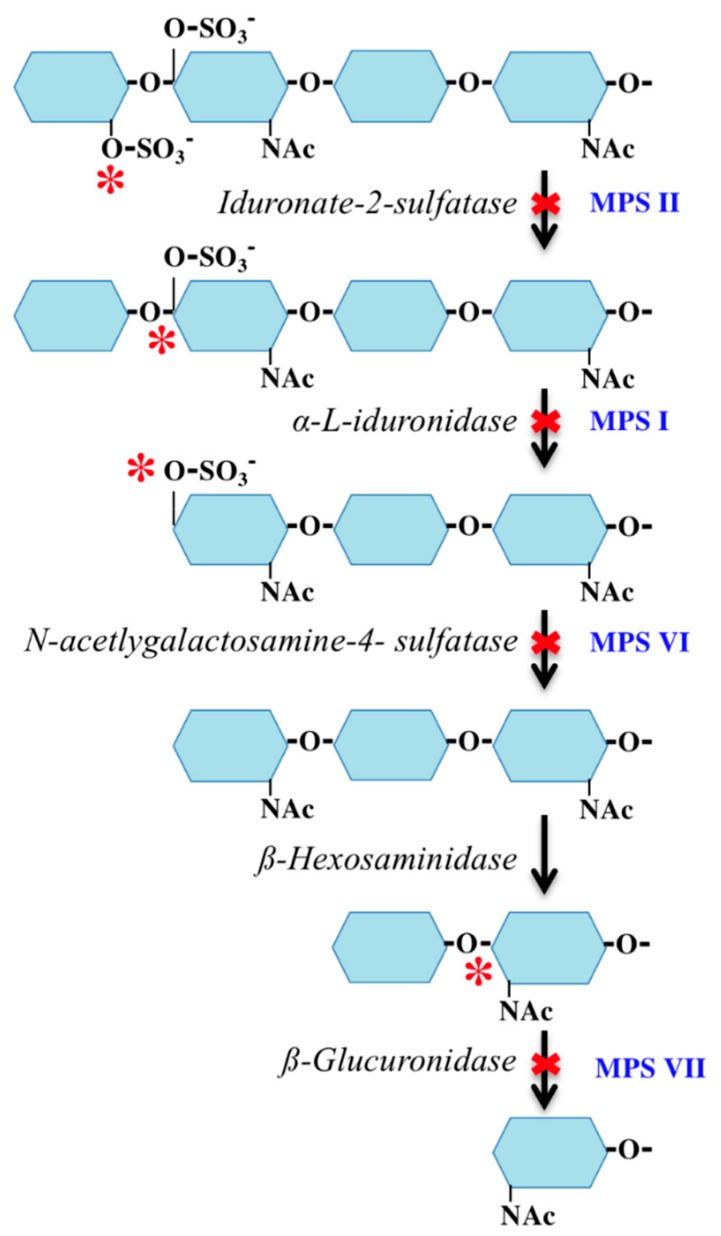
Dermatan sulfate (DS) degradation pathway. Enzymes are italicized. Disease process in blue. Asterisk indicates the location of the enzyme effect.

The deficient enzyme in MPS II (Figure 5) results in the build-up of DS containing an additional sulfate group (C-4 and C-2) as compared to MPS I and VI (C-4 only). We hypothesize that the additional sulfate group on the DS in MPS II exhibits a protective effect in the prevention of corneal clouding. There is support for this when comparing other pathologic disorders with reduced sulfation. Macular corneal dystrophy (MCD) is a severe form of corneal stromal dystrophy. The corneal clouding in this disorder is found to be due to the intracytoplasmic accumulations of non-sulfated KS [64]. Corneal transparency is dependent on the extent of sulfation of other GAGs, including DS. To determine the etiology of corneal clouding, detailed histopathological analyses and comparisons of intracytoplasmic accumulations of GAGs within the corneal stroma, endothelium, epithelium, and the Descemet membrane, as well as GAG compositions, will be required in different types of MPS.

## 14. Conclusions

Ocular management in MPS is challenging given the complexity of the condition in conjunction with limited available studies. As systemic therapies have increased the life expectancy of these patients, primary quality of life measures have renewed the focus into secondary measures, such as vision. Invasive ocular investigations and treatments may not be beneficial or necessary in patients who suffer from neurocognitive delays, as often seen in MPS III. However, patients without neurocognitive and behavioral delays may benefit from an eye-focused treatment like keratoplasty in addition to the systemic therapy. HSCT does have a higher mortality risk but can stabilize or improve corneal clouding. ERT, on the other hand, has lower risks but does not have any effects on the cornea. Future eye management will hopefully address the prevention of eye problems, such as corneal clouding, instead of treating patients after these have already set in.

## Figures and Tables

**Table 1:** Age of onset of ocular diseases in mucopolysaccharidosis (MPS). The ages presented in the table are earliest manifestations of ocular pathology reported in the literature on extensive case reviews. No meta-analysis was available aside from corneal clouding in MPS I. This table should only be used as a reference and not as a definitive guideline.

MPS Type (Eponym)	Enzyme Deficiency	Accumulated GAG	Inheritance	Incidence	Age of Onset (Years)			
					Corneal Clouding	Glaucoma	Retinopathy	Optic Neuropathy
MPS IH (Hurler)	α-l-iduronidase	HS, DS	AR	Attenuated: 1 in 115,000 to 500,000 live births	1.1	1	10	17
MPS IHS (Hurler-Scheie)	α-l-iduronidase	HS, DS	AR		4.4			
Hurler IS (Scheie)	α-l-iduronidase	HS, DS	AR	Severe: 1 in 100,000	10.5			
MPS II (Hunter)	Iduronate-2-sulfatase	HS, DS	XR	1 in 100,000 to 170,000 male births	N/A	7.5	<21	33
MPS IIIA (Sanfilippo A)	Heparan-*N*-sulfatase	HS	AR	1 in 70,000 live births	N/A	N/A	5	N/A
MPS IIIB (Sanfilippo B)	α-*N*-acetylglucosaminidase	HS	AR		N/A	N/A	N/A	N/A
MPS IIIC (Sanfilippo C)	α-glucosaminide acetyltransferase	HS	AR		N/A	N/A	N/A	N/A
MPS IIID (Sanfilippo D)	*N*-acetylglucosamine-6-sulfatase	HS	AR		8	N/A	N/A	N/A
MPS IVA (Morquio A)	*N*-acetylgalactosamine-6-sulfatase	KS, C6S	AR	1 in 76,000 to 640,000	11	7.8	12	N/A
MPS IVB (Morquio B)	β-galactosidase	KS						
MPS VI (Maroteaux-Lamy)	*N*-acetylgalactosamine-4-sulfatase	DS, C4S	AR	1 in 250,000 to 600,000 live births	7	3	N/A	26
MPS VII (Sly)	β-glucuronidase	DS, C4S, C6S, HS	AR	1 in 250,000 live births	15	N/A	N/A	N/A
MPS IX (Natowicz)	Hyaluronidase	Hyaluronan	AR	Unknown	N/A	N/A	N/A	N/A

Abbreviations: MPS—mucopolysaccharidosis; HS—heparan sulfate; DS—dermatan sulfate; KS—keratan sulfate; C6S—chondroitin-6-sulfate; C4S—chondroitin-4-sulfate; AR—autosomal recessive; XR—X-linked recessive; N/A—not applicable.

## Abbreviations

MPS	mucopolysaccharidosis
GAG	glycosaminoglycans
HSCT	hematopoietic stem cell transplantation
ERT	enzyme replacement therapy
DS	dermatan sulfate
HS	heparan sulfate
IOP	intraocular pressure
GALNS	*N*-acetylgalactosamine-6-sulfate sulfatase
KS	keratan sulfate
C6S	chondroitin-6-sulfate
C4S	chondroitin-4-sulfate
DALK	deep anterior lamellar keratoplasty
PK	penetrating keratoplasty
UMSC	umbilical mesenchymal stem cell
